# Blood levels of copeptin on admission predict outcomes in out-of-hospital cardiac arrest survivors treated with therapeutic hypothermia

**DOI:** 10.1186/cc11671

**Published:** 2012-10-04

**Authors:** Petr Ostadal, Andreas Kruger, Vladimira Zdrahalova, Marek Janotka, Dagmar Vondrakova, Petr Neuzil, Miroslav Prucha

**Affiliations:** 1 Department of Cardiology, Heart Center, Na Homolce Hospital, Prague 15030, Czech Republic; 2 Department of Clinical Biochemistry, Hematology, and Immunology, Na Homolce Hospital, Prague 15030, Czech Republic

## Abstract

**Introduction:**

Prognostic stratification of cardiac arrest survivors is essential for the selection of the most appropriate therapeutic strategy. However, accurate early outcome predictions for this patient population remain challenging. At present, there is a lack of data examining the prognostic value of C-terminal provasopressin (copeptin) in cardiac arrest survivors.

**Methods:**

A group of 40 out-of-hospital cardiac arrest survivors who were treated with endovascular hypothermia was analyzed. Copeptin levels were measured in blood samples taken at admission using a commercially available immunoassay. Neurological outcome was assessed at 30 days post admission according to the Cerebral Performance Category (CPC): CPC 1, no neurological deficit; CPC 2, mild to moderate dysfunction; CPC 3, severe dysfunction; CPC 4, coma; and CPC 5, death.

**Results:**

Copeptin levels were significantly lower in patients with CPC 1 compared with CPC 2 or CPC 3 to CPC 5 (74.3 ± 14.4 pmol/l, 219.8 ± 33.9 pmol/l and 302.7 ± 52.1 pmol/l, respectively; *P *< 0.0001). Using an optimal cutoff value ≤ 217.9 pmol/l calculated from the receiver operating characteristic curve (area under curve = 0.801, 95% confidence interval = 0.644 to 0.910; *P *= 0.0001), the sensitivity of predicting survival with good neurological outcome was 78.6% and the specificity was 75.0%. Multiple logistic regression analysis revealed that a copeptin level > 217.9 pmol/l was an independent predictor of severe neurological dysfunction or death, with an adjusted odds ratio of 27.00 (95% confidence interval = 2.27 to 321.68; *P *= 0.009).

**Conclusion:**

The present study found that copeptin levels have a significant prognostic value at the time of hospital admission, and are a promising diagnostic tool for predicting outcomes in out-of-hospital cardiac arrest survivors.

## Introduction

A large number of patients experience cardiac arrest each year, which is associated with high mortality and poor neurological outcomes [1,2]. In patients who survived to hospital admission, brain injury was the major cause of death; ischemic brain damage is responsible for approximately 70% of in-hospital deaths after out-of-hospital cardiac arrest [1,2]. Cerebral ischemia during cardiac arrest is also responsible for significant encephalopathy in many cardiac arrest survivors [1,2]. Before the introduction of therapeutic hypothermia into clinical practice, only 26 to 39% of patients survived cardiac arrest with good neurological outcomes - and even with all of the current therapeutic options, the proportion of cardiac arrest survivors with good neurological outcome remains as low as 49 to 55% [3,4]. Early risk stratification and outcome prediction are therefore essential for appropriate management modification and tailored resource allocation.

Several diagnostic tools have recently been shown to have prognostic value for cardiac arrest survivors who are treated according to contemporary recommendations, which include therapeutic hypothermia. Poor outcomes after cardiac arrest can be predicted by increased serum levels of neuron-specific enolase or protein S-100B, generalized background suppression or epileptiform complexes on electroencephalography, bilaterally absent cortical responses on somatosensory evoked potentials, and diffuse brain edema on computed tomography or magnetic resonance imaging [5]. To date, however, a reproducible and universally recognized prognostication method with a minimal false-positive rate is still not available for cardiac arrest patients.

Copeptin is the C-terminal fragment of the provasopresin peptide, and it is co-released from the hypothalamus, in an equimolar ratio, with the hypothalamic stress hormone vasopressin. Recently, it was shown that copeptin levels predict outcomes in several medical conditions, such as acute exacerbation of chronic obstructive pulmonary disease [6], ischemic stroke [7], myocardial infarction [8] and heart failure [9,10]. Data on the prognostic value of copeptin in cardiac arrest survivors are, however, still lacking. The objective of the present study was therefore to assess the utility of copeptin levels measured at admission in predicting outcome for out-of-hospital cardiac arrest survivors treated with endovascular hypothermia.

## Materials and methods

The present study was performed in accordance with the Declaration of Helsinki and the study protocol was approved by the Institutional Ethics Committee of the Na Homolce Hospital (Prague, Czech Republic). Surviving patients with favorable neurological outcomes and family members of deceased subjects or subjects with unfavorable neurological outcomes provided written informed consent retrospectively. Blood samples drawn from patients who were not willing to participate in the trial (which was expressed by family members in cases involving deceased subjects or subjects with unfavorable neurological outcomes) were discarded and the clinical data were not used in the analysis.

Patients who experienced cardiac arrest out of hospital with indications for mild therapeutic hypothermia (persistent coma with Glasgow Coma Score ≤ 8) were eligible to participate in the present study. Induction of hypothermia was initiated in the ambulance before hospital admission by infusion of ice-cold saline at a rate of 30 ml/kg/hour. Infusion continued after admission and was discontinued when the core temperature reached 34°C. An endovascular cooling method (Thermogard XP; Zoll, Chelmsford, MA, USA) with a target temperature of 33°C and a 24-hour hypothermia protocol was used in all patients [11]. Patients with ST-elevation myocardial infarction underwent direct percutaneous coronary intervention.

Blood samples for the measurement of copeptin levels were drawn at admission to the hospital (ICU or catheterization laboratory) before any interventional procedure. The serum was immediately separated by centrifugation at 1,500 rpm for 5 minutes and aliquots were stored at -70°C until measurement. Copeptin serum concentrations were assessed with the commercially available BRAHMS copeptin kryptor assay according to the manufacturer's instructions (BRAHMS, Hennigsdorf, Germany). The detection limit of the assay was 4.8 pmol/l, and the functional assay sensitivity was < 12 pmol/l. Lactate levels were measured routinely at hospital admission using a RAPIDPoint 500 analyzer (Siemens, Munich, Germany). Investigators performing the laboratory analyses were blinded to the clinical results.

Neurological outcome was assessed according to Cerebral Performance Category (CPC) [12] at 30 days post admission, which is widely used in research and quality assurance to assess neurological outcome following cardiac arrest: CPC 1, no neurological deficit; CPC 2, mild to moderate dysfunction; CPC 3, severe dysfunction; CPC 4, coma; and CPC 5, death. Physicians performing the CPC assessment were blinded to the copeptin levels.

The time to return of spontaneous circulation was defined as a period of time from collapse (obtained from witness) to restoration of spontaneous perfusing rhythm.

### Statistical methods

Continuous variables are presented as the mean ± standard deviation and compared using an unpaired *t *test and one-way analysis of variance with the Newman-Keuls multiple comparisons post test; categorical variables were presented as percentages and compared with Fisher's exact test. Receiver operating characteristic (ROC) curves were constructed to determine the optimal sensitivity and specificity of copeptin levels for predicting outcomes. A multiple logistic regression analysis model was applied to evaluate independent predictors of poor neurological outcome or death, and the prognostic value of copeptin levels. *P *< 0.05 was considered statistically significant. Statistical analyses were performed using GraphPad Prism version 5 (GraphPad Software, La Jolla, CA, USA) and MedCalc version 12 (MedCalc, Mariakerke, Belgium).

## Results

Forty-six patients were eligible to participate in the present study during the enrollment period (June 2010 to December 2011). One patient was excluded due to technical problems with blood-sample handling and five patients were excluded because written informed consent could not be obtained. In total, 40 patients (mean age 58 years) were included in the study, the majority of whom (32 of 40) were male (Table 1). Nineteen patients in the group survived without neurological dysfunction (CPC 1); nine patients survived with mild-to-moderate dysfunction (CPC 2); three survivors experienced severe neurological dysfunction (CPC 3 to 4); and nine patients died (CPC 5).

**Table 1:** Baseline characteristics

	CPC 1 to 2 (*n *= 28)	CPC 3 to 5 (*n *= 12)	*P *value	Odds ratio	95% CI
Age (years)	64.4 ± 12.4	69.3 ± 13.7	0.30		-14 to 4.5
Gender, male (%)	85.7	66.7	1.00	1.00	0.2 to 6.0
Diabetes (%)	46.4	41.7	1.00	1.21	0.3 to 4.8
Hypertension (%)	78.3	66.6	0.45	1.81	0.4 to 8.2
VFib (%)	71.4	75.0	1.00	0.83	0.2 to 3.9
Asystole (%)	21.4	25.0	1.00	0.82	0.2 to 4.0
AMI (%)	50.0	58.3	0.74	0.71	0.2 to 2.8
ROSC (min)	16.64 ± 9.34	22.82 ± 11.44	0.12		-14 to 1.7
LVEF (%)	35.87 ± 12.67	30.00 ± 15.67	0.26		-4.6 to 16
Lactate^a ^(mmol/l)	5.08 ± 3.19	8.25 ± 4.10	0.08		-6.7 to 0.4

Values expressed as the mean ± standard deviation unless otherwise indicated. ^a^Lactate level at admission. AMI, acute myocardial infarction; CI, confidence interval; CPC, Cerebral Performance Category; LVEF, left ventricular ejection fraction within 2 hours of admission; ROSC, return of spontaneous circulation; VFib, ventricular fibrillation.

**Figure 1:**
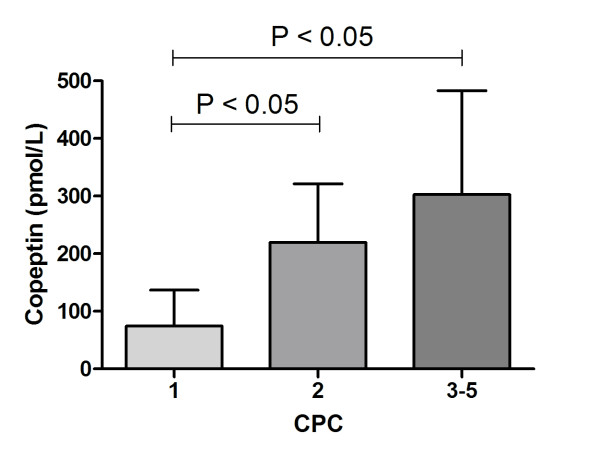
**Copeptin levels at hospital admission in cardiac arrest survivors according to Cerebral Performance Category**. Data presented as mean ± standard deviation. CPC, Cerebral Performance Category.

**Figure 2:**
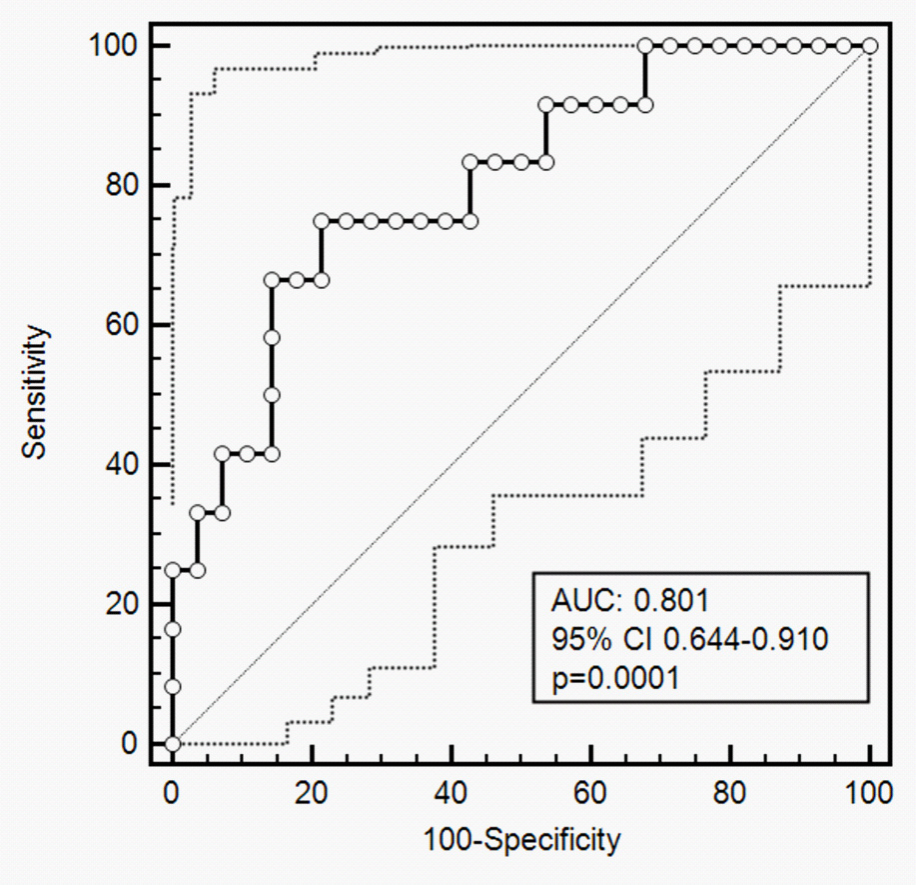
**Receiver-operating characteristic curve of copeptin for predicting outcomes in cardiac arrest survivors**. The optimal cutoff value for good neurological outcome (Cerebral Performance Categories 1 and 2) calculated from this receiver operating characteristic curve was ≤ 217.9 pmol/l, with sensitivity 78.6% and specificity 75.0%. Dotted lines: 95% confidence interval (CI) bounds. AUC, area under curve.

Copeptin levels were significantly lower in patients with CPC 1 compared with CPC 2 or CPC 3 to CPC 5 (74.3 ± 65.6 pmol/l, 219.8 ± 101.7 pmol/l and 302.7 ± 180.5 pmol/l, respectively; *P *< 0.0001) (Figure 1). The ROC curve was used to estimate the optimal cutoff level of copeptin for good neurological outcomes (CPC 1 and CPC 2; area under curve = 0.801, 95% confidence interval = 0.644 to 0.910; *P *= 0.0001) (Figure 2). Using an optimal cutoff value ≤ 217.9 pmol/l calculated from the ROC curve, the sensitivity of predicting survival with good neurological outcome was 78.6% and the specificity was 75.0% (Figure 2). Multiple logistic regression analysis was applied to determine whether the copeptin level was an independent predictor for poor neurological outcome or death. In the analysis, a copeptin level > 217.9 pmol/l was found to be an independent predictor, with an adjusted odds ratio of 27.00 (95% confidence interval = 2.27 to 321.68; *P *= 0.009) (Table 2).

**Table 2:** Multiple logistic regression analysis for independent predictors of poor neurological outcomes or death

Variable	Adjusted odds ratio	95% confidence interval	*P *value
Copeptin level > 217.9 pmol/l	27.00	2.2670 to 321.6817	0.0091
Myocardial infarction	6.26	0.4466 to 87.8582	0.1733
Ventricular fibrillation	0.85	0.0452 to 16.0444	0.9146
Age	1.07	0.9714 to 1.1701	0.1773
Return of spontaneous circulation	1.02	0.9419 to 1.1122	0.5831

## Discussion

The major finding of the present study was that the copeptin level, assessed at hospital admission, can predict neurological outcomes in cardiac arrest survivors. A cutoff value ≤ 217.9 pmol/l was determined by ROC curve analysis; patients with a copeptin level < 217.9 pmol/l were more likely to survive to 30 days without significant neurological dysfunction compared with patients with a copeptin level > 217.9 pmol/l. Early outcome prediction is challenging in cardiac arrest survivors, and the optimal timing as well as the methods used for prognostication remain controversial. Several approaches have been shown to have a prognostic value several days after cardiac arrest, such as assessment of serum markers of brain injury (neuron-specific enolase, S-100B), electroencephalography, somatosensory evoked potentials, and neuroimaging [5]. At the present time, however, only a very limited number of parameters can be used for prognosis prediction at the time of hospital admission after cardiac arrest. In this respect, including the measurement of copeptin levels among the screening laboratory investigations in these patients, using a widely available commercial assay may offer a relatively simple prognostication method.

The results of the present study are in agreement with other studies describing the prognostic value of copeptin levels in several other acute and/or critical conditions [13]. Copeptin levels have been recently shown to correlate with outcomes in the following conditions: acute exacerbation of chronic obstructive pulmonary disease [6], ischemic stroke [7], myocardial infarction [8,14], heart failure [9,10], and even in patients presenting to the emergency department with nonspecific complaints [15]. Moreover, Jarai and colleagues reported that preoperative copeptin concentrations predict postoperative outcome after major vascular surgery [16]. Our observations are also in good agreement with the study by Kim and colleagues, in which they described that cardiac arrest survivors with relative adrenal insufficiency, higher blood levels of adrenocorticotropic hormone and vasopressin have poor outcomes [17].

The present study, however, has several limitations mostly attributable to its limited sample size. Furthermore, all of the patients in our group were out-of-hospital cardiac arrest survivors who were treated with mild endovascular hypothermia, and it remains uncertain whether our results can be generalized to all cardiac arrest survivors, including patients who experience in-hospital arrest and those undergoing different cooling methods. Another possible limitation is that patients with chronic diseases that are known to be associated with elevation of copeptin such as heart or renal failure were not excluded.

## Conclusion

Our results demonstrate that copeptin level assessment at the time of hospital admission in cardiac arrest survivors can be useful in the prediction of neurological outcomes and mortality. Currently, a universal strategy for risk stratification after out-of-hospital cardiac arrest is still under development, and the use of copeptin measurement at admission may contribute significantly to improving both the speed and the accuracy of outcome prediction for this patient population.

## Key messages

• Copeptin level at admission predicts outcomes in out-of-hospital cardiac arrest survivors.

• Patients with a copeptin level < 217.9 pmol/l were more likely to survive to 30 days without significant neurological dysfunction.

## Abbreviations

CPC: Cerebral Performance Category; ROC: receiver operating characteristic.

## Competing interests

The authors declare that they have no competing interests.

## Authors' contributions

PO and MP were responsible for study conception and design. AK, DV and MJ were responsible for data acquisition, analysis, and interpretation. VZ and PO drafted the manuscript. PN and MP were responsible for critical revision of the manuscript. All authors read and approved the manuscript for publication.
